# From processing-centered innovation to analytical diversification: network evolution in virgin olive oil patents

**DOI:** 10.3389/frma.2026.1873036

**Published:** 2026-08-31

**Authors:** Turhan Karakaya, Sezai Tunca, Yavuz Selim Balcioglu

**Affiliations:** 1 Carbon Management Technician Program, Department of Environmental Management Technologies, Vocational School, Doğus University, Istanbul, Türkiye; 2 Department of Management, Faculty of Economics, Administrative and Social Sciences, Alanya University, Alanya, Türkiye; 3 Department of Management Information Systems, Faculty of Economics and Administrative Sciences, Doğus University, Istanbul, Türkiye

**Keywords:** innovation systems, analytical technologies, CPC co-classification networks, network modularity, patent families, patent landscape analysis, Salton normalization, technological diversification

## Abstract

**Introduction:**

This study examines how the technological structure of virgin olive oil (VOO) innovation evolved between 1990 and 2025. It asks whether the sector moved beyond its traditional processing base toward a more diversified innovation system, and whether analytical technologies became important connectors between technological domains.

**Methods:**

A patent landscape analysis was conducted on 811 deduplicated simple patent families retrieved from Lens.org. Cooperative Patent Classification (CPC) co-classification networks were constructed for three periods (1990–2004, 2005–2014, 2015–2025) and analyzed in terms of network size and density, Louvain community structure, technological-domain shares, and betweenness centrality. Robustness was assessed using Salton/cosine and Jaccard normalization and by excluding the incomplete final year.

**Results:**

The technological knowledge base expanded from 44 CPC subclasses and 72 co-classification ties to 86 subclasses and 328 ties. Modularity fell from 0.499 in 1990–2004 to 0.348 in 2005–2014 and then rose modestly to 0.376 in 2015–2025, a directional pattern reproduced under both normalizations. The analytical subclass G01N rose from 1.8% to 7.2% of CPC tokens and from 3.3% to 14.0% of patent families, and became a top-five bridging class in the recent period, while processing classes (C11B, A23L, A23D) remained the strongest full-period connectors. Within G01N, chromatographic and optical methods predominated.

**Discussion:**

VOO innovation follows a hybrid pattern of diversification, analytical intensification, and selective re-specialization, rather than a complete transition to a fully integrated bioactive platform. Measurement capability is an increasingly consequential complement to, but not a replacement for, processing competence.

## 1 Introduction

Virgin olive oil (VOO), and particularly extra virgin olive oil (EVOO), has long occupied a central position in the Mediterranean food system and global edible oil markets. Historically, innovation in this sector has been organized around the optimization of mechanical extraction processes, the maintenance of physicochemical quality parameters, and compliance with international classification standards. However, over the past two decades, a structural transformation has been unfolding. Driven by converging developments in phenolic bioactivity research, EU health claim regulation [European Food Safety Authority (EFSA), 2011; European Commission, 2012], advanced analytical chemistry, and sustainability imperatives, the innovation landscape of virgin olive oil has expanded well beyond its traditional processing core. EVOO is increasingly reconceptualized not merely as a commodity oil but as a bioactive platform whose value proposition depends on the generation, preservation, analytical verification, and credible communication of biologically active minor constituents.

Despite the maturity of individual research strands—processing science, phenolic bioactivity, advanced analytics, authenticity governance, and by-product valorization—an important gap remains at the systems level: these domains have largely advanced in parallel tracks, and no study has systematically examined whether and how they have become structurally integrated within the inventive knowledge base of the sector. Patent landscape analysis, combined with network methods applied to Cooperative Patent Classification (CPC) co-occurrence data, offers a uniquely suited approach for mapping the relational architecture of an innovation system and detecting structural transformations that are not visible in aggregate trend data alone (Leydesdorff, 2008; Boschma et al., 2015).

This study addresses the systems-level gap by analyzing 811 deduplicated simple patent families spanning 1990–2025. It examines how the technological architecture of VOO innovation expanded, how the relative positions of processing, pharmaceutical, sustainability, and analytical domains changed, and whether analytical technologies became important cross-domain connectors. Rather than assuming a predetermined transition toward an integrated platform, the analysis evaluates competing patterns of consolidation, diversification, and re-specialization.

Rather than presenting these questions in isolation, the study derives them systematically from the theoretical and empirical literature reviewed in Section 2. Section 2.1 situates the analysis within sectoral innovation systems and technological regime theory; Sections 2.2–2.6 review the domain-specific evidence on processing technology, health-claim regulation, analytical chemistry, and by-product valorization; and Section 2.7 identifies the resulting research gap and formally states the four research questions that guide the empirical analysis. In broad terms, the study asks how the technological structure of virgin olive oil innovation has diversified over time, whether its core has shifted from processing toward bioactive and nutraceutical applications, what role analytical technologies play in connecting otherwise separate domains, and whether the observed dynamics correspond to a specialization–reintegration cycle.

The remainder of the paper is organized as follows. Section 2 develops the theoretical framework, reviews the relevant literature, and derives the research questions. Section 3 describes the data, the construction and normalization of the CPC co-classification networks, and the analytical procedures. Section 4 reports the longitudinal, domain-level, network-structural, and robustness results. Section 5 discusses the theoretical, practical, and policy implications, and Section 6 concludes.

## 2 Literature review

### 2.1 Theoretical framework: sectoral innovation systems, technological regimes, and structural reconfiguration

The analysis presented in this paper is situated within the sectoral systems of innovation perspective, which conceptualizes an industry as a system composed of a distinctive knowledge base, a population of heterogeneous actors, and a set of institutions that shape the direction and rate of technical change (Malerba, 2002). A central implication of this perspective is that sectoral development cannot be adequately described by aggregate measures of inventive output alone. What changes over time is not only how much innovation occurs, but how the constituent knowledge elements are combined, which competencies are drawn together, and which technological domains become mutually relevant. Structural change therefore refers to a reorganization of the relational architecture of the knowledge base, and it is this reorganization, rather than volumetric growth, that the present study seeks to identify and measure.

Evolutionary approaches to technical change specify the mechanisms through which such reorganization occurs. Technological development is understood as a cumulative and path-dependent search process conducted within a technological regime, that is, a relatively stable configuration of engineering practices, search heuristics, and selection criteria that channels the direction of inventive activity (Nelson and Winter, 1982). Regimes are not immutable: when new scientific evidence, regulatory requirements, or market expectations enter the system, previously peripheral activities may accumulate into niches that eventually destabilize and reconfigure the incumbent regime (Geels, 2002). Reconfiguration, importantly, differs from replacement. In a reconfiguration pathway the established core is not displaced but absorbed into a broader architecture in which additional functional domains become structurally embedded. Diversification and recombination are the elementary operations of this process: new variety is generated by combining knowledge elements that were previously unconnected, and the feasibility of such combinations depends on the relatedness of the domains involved (Saviotti and Frenken, 2008; Boschma et al., 2015).

Two further theoretical strands inform the interpretation advanced here. The first concerns platform logics. Platform theory, developed primarily with reference to digital industries, describes architectures in which a stable core enables a variety of complementary applications and thereby supports multi-actor value creation (Gawer and Cusumano, 2014). Whether comparable logics operate in traditional agri-food sectors, and what performs the coordinating function in the absence of software interfaces and network effects, remains an open question. The second strand concerns the economics of standards and metrology, which demonstrates that measurement conventions, testing procedures, and certification infrastructures are not neutral downstream instruments but constitutive elements of markets and of innovation trajectories: they determine what can be claimed, compared, and traded, and consequently what is worth inventing (Blind, 2004). Taken together, these strands suggest that in sectors where product value depends on compositional properties that are invisible to the user, measurement infrastructures may perform the coordinating function that interfaces perform in digital platforms. This proposition is the conceptual point of departure for the empirical analysis reported below.

Empirically, patent documents and their classification codes provide a well-established window on the architecture of a sectoral knowledge base. Because classification codes are assigned by examiners according to a common hierarchical scheme, the co-assignment of two codes to the same invention constitutes an observable trace of knowledge recombination, and the resulting co-classification network can be read as an indicator of the intellectual organization of a technological field (Leydesdorff, 2008). Combining patent big data with social network analysis has accordingly become an established strategy for reconstructing the spatio-temporal evolution of industrial technologies, identifying technical clusters, and detecting cross-boundary integration mechanisms within innovation ecosystems. Recent applications illustrate the versatility of the approach across very different sectoral contexts. In fusion engineering, a hybrid topic-modeling and network framework has been used to trace the displacement of research effort from physics validation toward engineering realization and to characterize the division of labor among institutional actors (Cai et al., 2026). In the ready-to-cook food industry, a combined space–time–technology–network framework has been applied to map technical clustering and collaborative structures in a rapidly emerging agri-food segment (Cai et al., 2025). In the electric vehicle sector, network indicators such as density, transitivity, clustering, and component structure have been used to assess the coordination of policy systems and its relationship to market outcomes (Wang et al., 2025). The present study adopts a comparable analytical logic while shifting the emphasis from actor collaboration to the internal architecture of the knowledge base, and from a single period to a longitudinal comparison of successive structural configurations.

### 2.2 From processing quality to bioactivity: the reorientation of the olive oil knowledge base

The subsections that follow review the domain-specific literature documenting this reorientation. For readers approaching the topic from innovation studies rather than food science, the essential point is not the chemistry itself but its systemic consequence. As the value of the product came to depend on constituents that are present in minute quantities and are imperceptible to the consumer, the innovation system acquired a new functional requirement: the capacity to generate, preserve, verify, and credibly communicate those constituents. Each of the following subsections examines one component of that requirement and draws out its implications for the structure of the knowledge base. Comparable reorientations toward health-oriented and functional product categories have been documented across the wider food industry (Bigliardi and Galati, 2013), and the ability of firms in mature sectors to absorb and recombine externally generated knowledge is a recognized determinant of their innovation performance (Ferraris et al., 2020).

Virgin olive oil (VOO), and particularly extra virgin olive oil (EVOO), has historically been investigated through a processing- and quality-oriented paradigm, where research primarily focused on mechanical extraction systems, physicochemical stability, sensory characteristics, and regulatory standards used to classify olive oils in international markets. However, during the past decade, the scientific and innovation discourse surrounding EVOO has undergone a significant conceptual transformation. Rather than being considered solely as a lipid product derived from optimized mechanical processing, EVOO is increasingly conceptualized as a bioactive platform, whose value proposition depends on the generation, preservation, analytical verification, and credible communication of biologically active minor constituents. This shift reflects the convergence of several structural developments in olive oil science. First, a growing body of biomedical and nutritional research has demonstrated that olive oil phenolic compounds—particularly hydroxytyrosol, tyrosol, and their derivatives—exhibit strong antioxidant activity and contribute to cardiovascular protection by reducing oxidative stress and inflammation (Covas et al., 2006). These findings were formally acknowledged by the [European Food Safety Authority (EFSA), 2011], which concluded that a cause-and-effect relationship exists between the consumption of olive oil polyphenols and the protection of low-density lipoprotein (LDL) particles from oxidative damage. As a consequence, the European regulatory framework authorized a health claim indicating that olive oils containing at least 5 mg of hydroxytyrosol and its derivatives per 20 g of oil may communicate this protective effect to consumers (European Commission, 2012). The institutionalization of this health claim has substantially increased the demand for reliable analytical methodologies capable of accurately quantifying phenolic compounds and ensuring transparency in product labeling and market competition (Mastralexi et al., 2014). In parallel, advances in analytical chemistry—particularly the development of high-resolution metabolomic and spectroscopic techniques such as nuclear magnetic resonance (NMR) spectroscopy and mass spectrometry—have dramatically expanded the ability to characterize the complex molecular composition of olive oil and generate chemical fingerprints associated with cultivar, geographical origin, and processing conditions (Dais and Hatzakis, 2013). Collectively, these developments have reconfigured the epistemic foundations of olive oil research, transforming EVOO from a simple processed agricultural commodity into a multidimensional biochemical system in which process engineering, phenolic bioactivity, regulatory governance, and advanced analytical infrastructures interact to shape the technological trajectory and market value of olive oil innovation.

### 2.3 Regulation as an institutional driver: the European health-claim framework

In innovation-system terms, the regulatory development described in this subsection operates as a selection mechanism. By attaching a legally recognized benefit to a quantified compositional threshold, the European framework converted a diffuse nutritional narrative into a specific and measurable technological target, and thereby redefined the criteria against which inventive activity in the sector is evaluated.

A key catalyst in this reorientation has been the institutionalization of health-claim logic within the European regulatory framework. The scientific opinion issued by the European Food Safety Authority established an evidence-based relationship between the intake of olive oil polyphenols and the protection of blood lipids from oxidative stress, thereby linking the phenolic composition of olive oil to a clearly defined physiological outcome (European Food Safety Authority (EFSA), 2011). This scientific assessment was subsequently translated into regulatory practice through Commission Regulation (EU) No 432/2012, which formally authorized the health claim and specified the conditions under which it may be used (European Commission, 2012). According to this framework, the claim can only be applied to olive oils providing at least 5 mg of hydroxytyrosol and its derivatives per 20 g of oil, with the beneficial effect associated with that daily intake. By codifying this biochemical threshold, the European regulatory architecture effectively transforms “bioactivity” from a general nutritional narrative into a measurable technological target. Consequently, innovation activities increasingly focus on managing phenolic composition throughout the production chain—from cultivation and harvest timing to processing conditions and storage stability—in order to achieve and maintain compliance with the regulatory threshold. At the same time, the framework elevates analytical reliability to a central component of the innovation system, since reproducible and validated methods for quantifying hydroxytyrosol-related phenolics become essential for substantiating the claim and stabilizing the bioactive value proposition of olive oil in the market.

### 2.4 Processing as bioactive-preservation engineering

The literature summarized here establishes that the phenolic profile of the finished product is largely determined by processing choices rather than fixed by the raw material. The systemic implication is that the traditional processing core of the sector was not rendered obsolete by the turn toward bioactivity. It was instead reassigned a new function within the emerging architecture, becoming the principal lever through which newly valorized compositional targets are attained.

Within the contemporary understanding of extra virgin olive oil production, processing is increasingly interpreted not merely as a mechanical extraction stage but as a complex technological environment that determines the composition and stability of the phenolic fraction. Rather than being passive constituents transferred unchanged from fruit to oil, phenolic compounds are dynamic molecules whose concentration and chemical forms are influenced by enzymatic reactions, oxidation processes, and phase partitioning mechanisms occurring during crushing, malaxation, separation, and subsequent storage (Servili et al., 2003). Consequently, research has progressively shifted from optimizing individual processing parameters toward a more systemic management of the extraction environment. Operational factors such as malaxation time and temperature, oxygen exposure, water addition, separation technology, filtration, and storage conditions interact to shape both the total phenolic content and the relative abundance of key hydroxytyrosol- and tyrosol-derived compounds that contribute to the nutritional and sensory characteristics of olive oil (Kalua et al., 2007; Boskou, 2015). In this sense, processing can be conceptualized as a form of bioactive-preservation engineering, where technological choices are strategically configured to maintain the phenolic profile that underpins both the functional value and the distinctive sensory identity of high-quality olive oil.

The bioactive-platform framing is strengthened by biomedical and nutritional evidence showing that olive phenolics (notably hydroxytyrosol, tyrosol, oleuropein derivatives, and related secoiridoids such as oleocanthal/oleacein) participate in antioxidant and anti-inflammatory pathways plausibly relevant to cardiometabolic risk biology (Filardo et al., 2024; Karkovic Markovic et al., 2019). Crucially, this literature also converges on a *pharmacology-like* insight: what matters is not mere “presence” of phenolics in the food matrix, but exposure, i.e., bioavailability, metabolism, and dose—because these determine whether biologically active metabolites appear in circulation and at what magnitude (de Bock et al., 2013; Karkovic Markovic et al., 2019). Consistent with that exposure logic, controlled human interventions using olive oils that differ mainly in phenolic content have reported dose-dependent shifts in oxidative stress-related markers alongside increases in urinary tyrosol/hydroxytyrosol, supporting the idea that phenolic composition can translate into measurable biological signals when intake is sufficient and sustained (Marrugat et al., 2004). For innovation studies, the implication is that value creation in EVOO increasingly depends on minimizing ‘slippage' across three linked layers—composition → exposure → claimable benefit—which, in turn, makes analytical integration (credible quantification, exposure characterization, and documentation) not a technical afterthought but the coordinating infrastructure of the platform proposition.

### 2.5 Analytical integration as a coordinating infrastructure

This subsection develops the argument that is central to the present study. Where value depends on quantified thresholds, the measurement infrastructure ceases to be a downstream control function and becomes the mechanism through which otherwise separate domains—process engineering, health-related formulation, authenticity governance, and sustainability—are rendered mutually legible and combinable. This proposition, derived here from the literature, is tested empirically in Section 4 through the bridging position of analytical classifications within the co-classification network.

Analytical integration refers to more than the adoption of advanced instruments; it denotes a structural coupling between measurement regimes and the governance of quality, authenticity, and innovation trajectories. The rise of NMR spectroscopy and mass spectrometry-based metabolomics has transformed EVOO from a product evaluated by a small set of routine indices into a chemically “high-dimensional” object, where both targeted and untargeted profiles are used for classification, origin attribution, adulteration detection, and compositional benchmarking. The canonical critical review by Dais and Hatzakis (2013) positions NMR not simply as an analytical alternative, but as a platform technology enabling compositional fingerprinting, chemometric discrimination, and authenticity workflows—capabilities that are increasingly central in premium markets vulnerable to mislabeling and fraud. Complementing this, more recent work using untargeted LC-qToF/LC-HRMS demonstrates how high-resolution fingerprints and multivariate models can detect adulteration and differentiate oils on the basis of subtle chemical markers, extending the authenticity frontier beyond what targeted panels can reliably capture. These developments collectively signal a shift from “quality control” as downstream inspection to “quality intelligence” embedded in the value chain, with chemometrics functioning as the interpretive layer that converts spectra and feature tables into actionable classifications.

Crucially, the analytical turn is inseparable from the health-claim regime. Once bioactivity becomes claimable only through quantified thresholds, method selection, validation, and inter-laboratory reproducibility become innovation bottlenecks. The analytical requirements to support the EU health claim on olive oil polyphenols have been explicitly articulated as a scientific and operational challenge: how to measure hydroxytyrosol and its derivatives robustly enough to underpin regulatory compliance and credible labeling (Mastralexi et al., 2014). This line of inquiry situates analytical chemistry as part of the institutional infrastructure of markets—an enabling condition for bioactive differentiation, not a peripheral technical detail. In innovation-system terms, the laboratory, the standard, the model, and the label form an integrated socio-technical assemblage: compositional measurement stabilizes claims; claims reshape production incentives; incentives drive process redesign and cultivar/harvest strategies; and these, in turn, alter the chemical space that analytics must resolve.

### 2.6 Boundary expansion: by-product valorization and the circular bioeconomy

A further indication of structural change concerns the boundaries of the system itself. As the following discussion shows, material flows previously classified as waste have been redefined as inputs, drawing new competencies and actors into the sector and extending the range of outputs over which innovation is organized.

The “structural reconfiguration” highlighted in the research theme becomes most visible when the EVOO value chain is examined beyond the primary product. A platform logic tends to generate multiple valorization pathways, and olive oil production is characterized by substantial side streams (e.g., pomace, olive mill wastewater) that are rich in phenolics and other compounds. As a result, what was historically treated as waste or an environmental liability is increasingly reframed as a resource base for extraction, recovery, and downstream applications in nutraceutical, cosmetic, and functional ingredient markets. This is a classic signature of structural reconfiguration: the innovation system expands its boundary conditions, pulls in new actors and competencies (separation technologies, green chemistry, bio-refining, regulatory expertise), and reorganizes around multi-output value creation rather than a single commodity. The circular bioeconomy framing here is not merely sustainability rhetoric; it changes the locus of innovation and the portfolio of profitable R&D targets, thereby reshaping the knowledge network that underpins the sector.

### 2.7 Research gap and derivation of the research questions

Despite the maturity of each individual strand—processing science, phenolic bioactivity, advanced analytics, authenticity, and by-product valorization—an important gap remains at the level implied by the title: their integration as a coherent innovation architecture. The literature often advances in parallel tracks: process studies optimize conditions but do not always translate phenolic shifts into claim-relevant metrics; metabolomics studies classify origins or detect adulteration but may remain weakly connected to process causality and regulatory thresholds; and health-oriented reviews may not map back onto controllable process levers or analytically reproducible exposure measures. The platform thesis therefore calls for an explicitly integrative research agenda that treats EVOO innovation as a coupled system in which (i) process variables are modeled as drivers of (ii) compositional and fingerprint-level changes, which are then linked to (iii) claim-relevant quantification and authenticity assurance, under (iv) institutional and market constraints that determine which bioactive profiles are economically and legally meaningful.

In sum, the contemporary EVOO literature suggests that processing, bioactivity, regulation, analytical validation, and by-product valorization have become increasingly interdependent. Whether this interdependence produces a single integrated platform, a set of specialized technological modules, or a hybrid architecture remains an empirical question. The patent-network analysis therefore treats structural change as an open outcome rather than presupposing a specialization–reintegration cycle.

The literature yields four observational expectations. First, technological diversification should appear as growth in the number of CPC subclasses and co-classification ties. Second, a changing balance between processing and pharmaceutical classifications should reveal whether the sector's inventive emphasis has shifted. Third, if analytical technologies coordinate otherwise separate domains, G01N should gain both prevalence and bridging centrality, although its position must be tested against normalized networks. Fourth, the direction of modularity change should indicate whether the system is consolidating, fragmenting, or selectively re-specializing over time.

RQ1. How has the technological structure of virgin olive oil innovation evolved between 1990 and 2025 in terms of diversity, connectivity, and network scale?

RQ2. How have the relative positions of processing, pharmaceutical/nutraceutical, analytical, and sustainability-related technologies changed over time?

RQ3. To what extent has analytical chemistry (G01N) become a cross-domain bridge, and is this position robust to Salton/cosine and Jaccard normalization?

RQ4. Does the longitudinal modularity pattern indicate consolidation, fragmentation, reintegration, or selective re-specialization of the innovation system?

## 3 Methodology

### 3.1 Data source and retrieval strategy

Patent data were retrieved from the Lens.org database, which aggregates global patent documents across major jurisdictions including the USPTO, EPO, WIPO, and national offices. Lens.org was selected for its comprehensive coverage, open-access availability, and integrated deduplication features, which facilitate large-scale patent landscape analyses (Jefferson et al., 2018).

The search strategy targeted patent records related to virgin and extra virgin olive oil in food, processing, analytical, packaging, health-related, and adjacent technological contexts. The exported fields included publication year, jurisdiction, title, abstract, applicants, Lens ID, Simple Family Members, and complete CPC classifications. Cosmetic-only and biodiesel-only records were screened where identifiable from titles, abstracts, and classifications.

The retrieval was designed to identify a technologically relevant VOO corpus rather than an exhaustive census of every invention mentioning olive oil. Patent terminology and family-level metadata can vary across jurisdictions, and some relevant inventions may describe olive oil generically or confine grade-specific wording to claims. The resulting dataset should therefore be interpreted as a reproducible analytical corpus defined by the Lens export and subsequent filtering rules.

The Lens export contained 1,993 patent-document records. Simple patent families were reconstructed using the canonicalized Simple Family Members field, which prevents family-size counts from being mistaken for family identifiers. After family-level deduplication, restriction to 1990–2025, and removal of observations without usable CPC information, the final analytical dataset comprised 811 unique patent families.

For each patent family, the following variables were extracted: publication year, jurisdiction, applicant names, and Cooperative Patent Classification (CPC) codes assigned at the time of publication.

### 3.2 Data cleaning and standardization

All analyses were conducted at the patent family level to ensure that each unique invention was counted once, regardless of the number of jurisdictional filings.

CPC codes were parsed at the four-character subclass level (e.g., A23L, A61K, G01N). The final dataset contained an average of 1.961 distinct CPC subclasses per patent family. This level retains interpretable technological domains while limiting fragmentation. For the G01N subset, a second pass parsed main-group codes such as G01N 30/00 and G01N 33/00.

The four-character subclass level is used for all network construction and for the domain-level analyses reported in Section 4. In addition, and in order to resolve the internal heterogeneity of the G01N subclass, a second parsing pass was performed at the main group level (that is, at the level of codes of the type G01N 33/00) for the subset of families carrying at least one G01N code. The rationale, procedure, and aggregation scheme for this finer-grained analysis are set out in Section 3.7.

### 3.3 Temporal segmentation

The dataset was divided into three fixed analytical windows to permit longitudinal comparison:

- 1990–2004 (early processing-centered configuration): 121 patent families.
- 2005–2014 (network consolidation and domain broadening): 225 patent families.
- 2015–2025 (analytical diversification and selective re-specialization): 465 patent families.

The period labels are descriptive interpretations of the observed network structures rather than assumptions imposed before analysis. The same boundaries are retained to enable direct comparison with the original analytical design and with broader changes in functional-food and nutraceutical innovation.

Because patent applications are ordinarily disclosed after a lag, 2025 is treated as an incomplete year. It is retained in the primary period for transparency, but all third-period indicators were recomputed for 2015–2024. The truncated sample contains 433 families, 83 nodes, and 316 edges, and produces only small changes in density and modularity.

### 3.4 Technological domain classification

To examine thematic shifts across periods, patent families were categorized into broad technological domains using CPC-based mapping. The following domain groupings were defined:

- Food processing: classes associated with food preparation, edible oils, and related manufacturing processes (e.g., A23^*^, A21^*^).
- Pharmaceutical and nutraceutical formulations: classes covering medicinal preparations and therapeutic activity (A61K, A61P).
- Analytical and validation technologies: classes related to chemical and physical testing and measurement (G01N).
- Packaging and materials: classes encompassing container design, polymeric materials, and forming technologies (B65D, C08^*^, B29^*^).
- Digital and traceability technologies: classes related to computing, data processing, and communication systems (G06^*^, H04^*^).

Domain presence was measured at the patent family level using binary indicators (present/absent per family), enabling calculation of domain shares and their evolution across the three temporal windows.

### 3.5 Network construction

Technological structure was analyzed through CPC co-classification networks, a method widely used in patent-based innovation studies to map knowledge linkages and technological convergence (Leydesdorff, 2008; Boschma et al., 2015). In these networks, nodes represent four-character CPC subclasses and edges represent the co-occurrence of two CPC classes within the same patent family. Edge weights reflect the frequency of co-occurrence across all families within a given period.

Raw co-occurrence weights can privilege frequent subclasses. The co-occurrence matrix was therefore additionally normalized using the Salton/cosine index, $S\left(i,j\right)=c\left(i,j\right)/\sqrt{c\left(i\right)*c\left(j\right)}$, and the Jaccard index, $J\left(i,j\right)=c\left(i,j\right)/\sqrt{c\left(i\right)+c\left(j\right)-c\left(i,j\right)}$. Here *c*(*i*,*j*) is the number of families containing both subclasses, and *c*(*i*) and *c*(*j*) are their marginal family frequencies.

Node and edge counts and density are invariant to edge-weight normalization, whereas weighted modularity, clustering, and shortest-path-based betweenness can change. Results are consequently reported for raw, Salton/cosine, and Jaccard specifications. Rank correlations assess the degree of agreement among centrality measures rather than assuming that a frequent class is necessarily a structural bridge.

Separate networks were constructed for each of the three temporal windows to enable longitudinal structural comparison. This approach captures not only the expansion of the technological base but also the evolving architecture of inter-domain linkages.

### 3.6 Network metrics

To assess structural transformation across periods, the following network metrics were computed:

Structural indicators included the number of nodes (representing technological diversity), the number of edges (capturing cross-domain interactions), and network density (measuring overall connectivity as the ratio of observed to possible ties).

Community structure was assessed using modularity computed via the Louvain method (Blondel et al., 2008). Rising modularity indicates increasing segmentation into specialized clusters, while declining modularity suggests reintegration of previously distinct domains.

Centrality measures included degree centrality, which identifies the most connected technological domains, and betweenness centrality, which identifies bridging technologies that connect otherwise separate clusters. Betweenness centrality was of particular analytical importance for detecting integrative domains that serve as structural connectors within the innovation system.

### 3.7 Fine-grained analysis of analytical technologies

G01N is a broad subclass covering multiple analytical and testing methods. To determine whether its recent growth reflects compositional analysis rather than generic testing, all G01N families were disaggregated at the main-group level. The analysis separately reports chromatography (G01N 30/00), optical and spectrophotometric methods (G01N 21/00), chemical or biological property determination (G01N 33/00), magnetic-resonance methods (G01N 24/00), electrochemical methods (G01N 27/00), and remaining G01N groups.

Main-group categories are not mutually exclusive because a patent family can carry several G01N main groups. Shares are therefore calculated against both the 58 G01N families and all 433 patent families in the 2015–2024 truncated window, and their percentages should not be summed to 100%.

### 3.8 Operationalizing structural reconfiguration

Structural evolution was operationalized through changes in technological diversity (nodes), recombination (edges), density, modularity, domain shares, and bridging centrality. The interpretation distinguishes network growth from consolidation or fragmentation: a larger network can become more or less modular depending on how new links are distributed.

This multi-indicator design allows the empirical pattern to determine whether the sector exhibits integration, re-specialization, or a hybrid configuration rather than treating structural reconfiguration as synonymous with declining modularity.

### 3.9 Software and reproducibility

All data processing and network analyses were conducted in Python using pandas, NetworkX, SciPy, and custom scripts. Louvain communities were computed with a fixed random seed for reproducibility. Network visualizations used common layouts across periods so that recurring CPC classes occupy comparable positions. The cleaned family-level dataset, analysis scripts, and generated tables are retained by the corresponding author. An overview of the retrieval, deduplication and filtering sequence that produced the final analytical corpus is provided in Supplementary Figure S1.

### 3.10 Analytical limitations

Several limitations should be acknowledged. Patent data reflect inventive activity and formal knowledge claims rather than market adoption or commercial diffusion, and may therefore overrepresent jurisdictions with stronger patenting cultures and underrepresent innovation in regions where informal knowledge transfer predominates. CPC classification depends on examiner assignment and may not fully capture emergent or informal technological convergence that has not yet been codified in patent documentation.

Additionally, the use of four-character CPC codes, while appropriate for domain-level analysis, may mask finer-grained distinctions within broad classes such as A61K, which encompasses diverse formulation types. Finally, the temporal segmentation, although informed by preliminary data analysis and existing literature, involves boundary choices that could influence the comparative findings.

The retrieval and family-reconstruction process also imposes limitations. The final corpus contains 811 CPC-usable simple families from 1,993 exported documents, and differences in Lens indexing, family membership, or search-field behavior can alter counts across download dates. The study therefore reports the exact export and filtering sequence and does not treat the corpus as an exhaustive census. The 2025 endpoint is incomplete; robustness results for 2015–2024 are reported explicitly.

These limitations are mitigated by the longitudinal and network-based analytical design, which focuses on structural patterns and relational dynamics rather than the classification of individual documents.

## 4 Results

### 4.1 Sample and network expansion (1990–2025)

**Figure 1:**
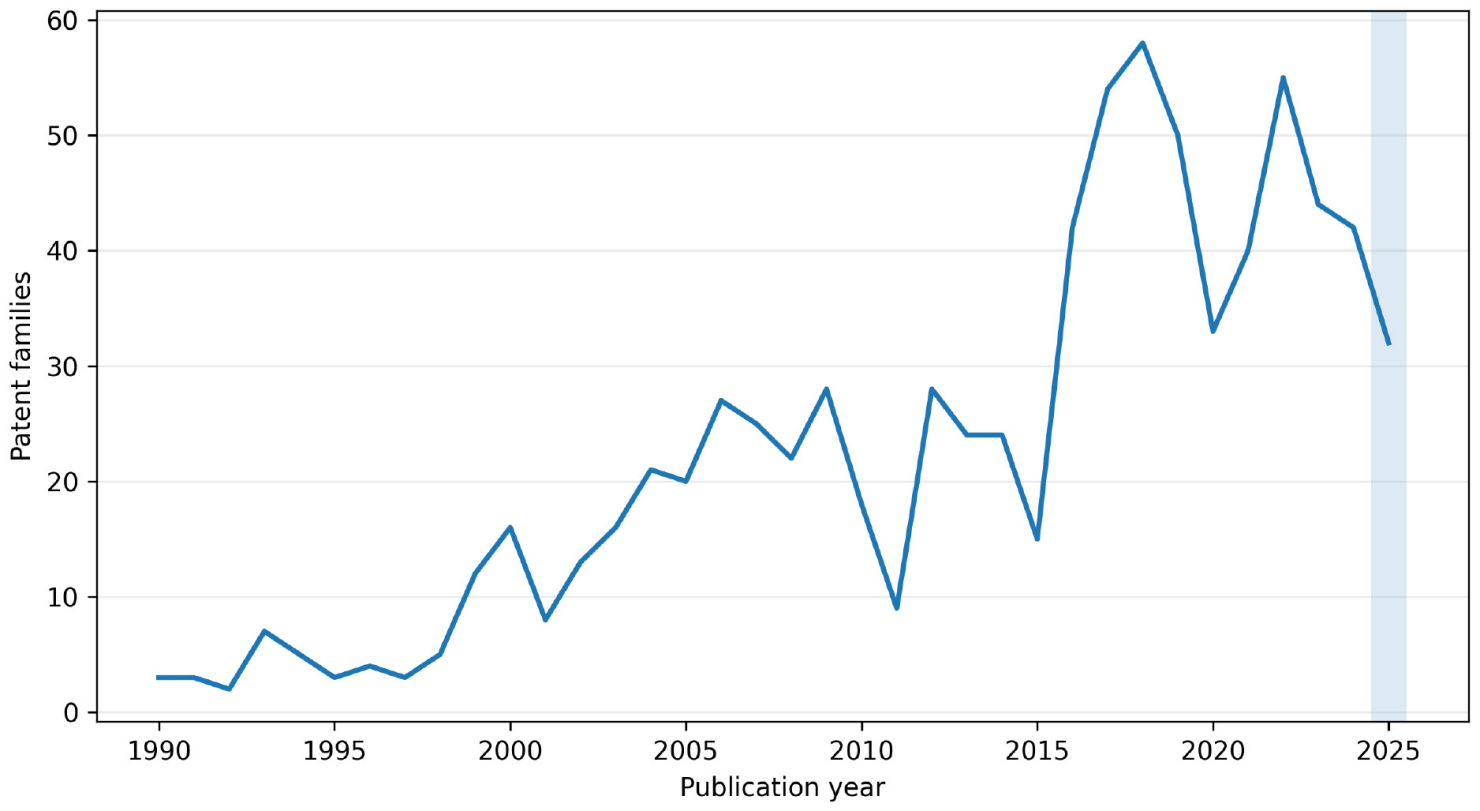
Annual patent family counts (1990–2025). The shaded final year is incomplete and is not interpreted as a substantive decline.

The reconstructed analytical corpus contains 811 simple patent families: 121 in 1990–2004, 225 in 2005–2014, and 465 in 2015–2025. Annual counts rise markedly over the observation window, although the final year is incomplete because of patent-publication lag (Figure 1).

**Figure 2:**
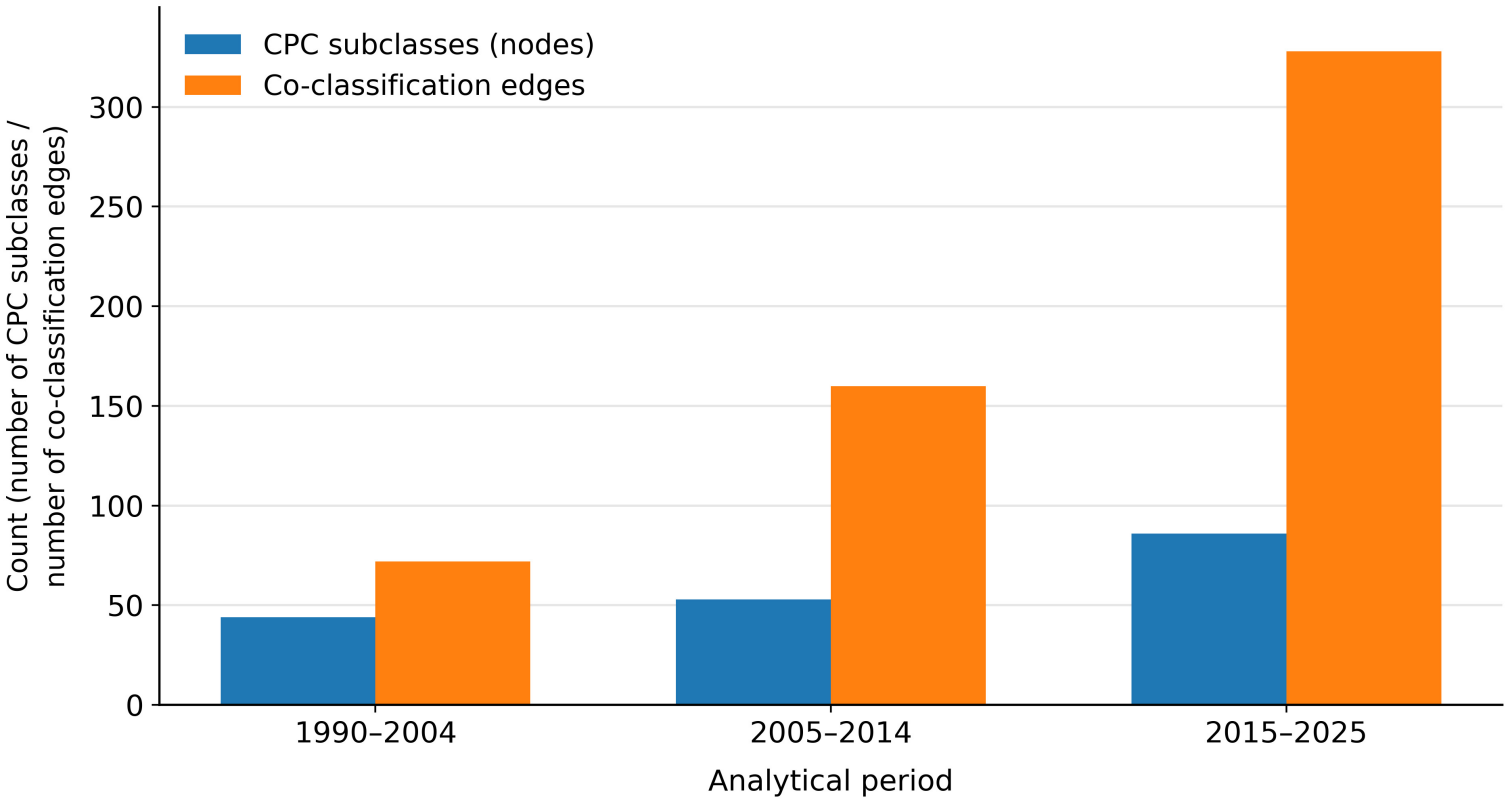
Technological diversity and cross-domain linkages across periods.

Technological diversity and recombination increased strongly. The network expanded from 44 CPC subclasses and 72 co-classification edges in the first period to 53 nodes and 160 edges in the second, and to 86 nodes and 328 edges in the third. The number of nodes therefore almost doubled and the number of observed ties increased more than four-fold (Figure 2; Table 1). Density rose from 0.076 to 0.116 between the first two periods and then declined to 0.090, indicating that the middle period was comparatively consolidated whereas the recent system expanded faster than its realized pairwise connectivity.

**Table 1:** Longitudinal network structure metrics.

Period	Families	Nodes	Edges	Density	Raw modularity
1990–2004	121	44	72	0.076	0.499
2005–2014	225	53	160	0.116	0.348
2015–2025	465	86	328	0.090	0.376

### 4.2 Consolidation followed by selective re-specialization

**Figure 3:**
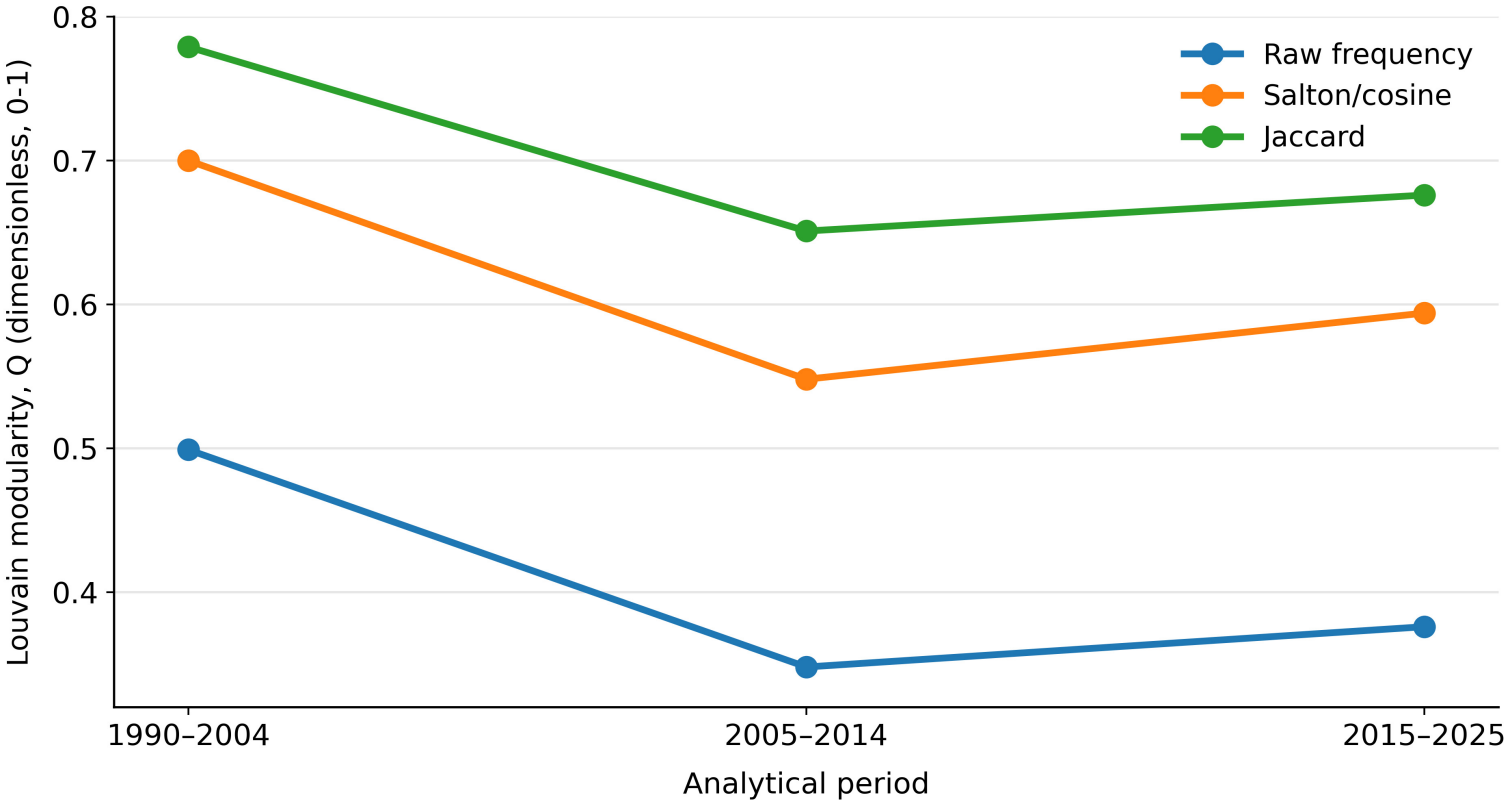
Louvain modularity (Q) of the CPC co-classification networks across the three analytical periods, under raw, Salton/cosine, and Jaccard edge weighting.

The modularity results do not reproduce the originally hypothesized specialization–reintegration cycle. Raw modularity fell from 0.499 in 1990–2004 to 0.348 in 2005–2014, indicating that previously separated technological regions became more connected during the middle period. It then increased modestly to 0.376 in 2015–2025. Salton/cosine modularity followed the same directional pattern (0.700, 0.548, and 0.594), as did Jaccard modularity (0.779, 0.651, and 0.676) (Figure 3).

This trajectory is best interpreted as early fragmentation, subsequent network consolidation, and recent selective re-specialization within a much larger technological space. The recent rise in modularity is modest relative to the first-period level and occurs alongside substantial growth in nodes and edges. Thus, the system has not returned to its early configuration; instead, new analytical, sustainability, and adjacent technologies have formed partially differentiated modules around a still-connected processing core.

### 4.3 Domain dynamics and analytical intensification

**Figure 4:**
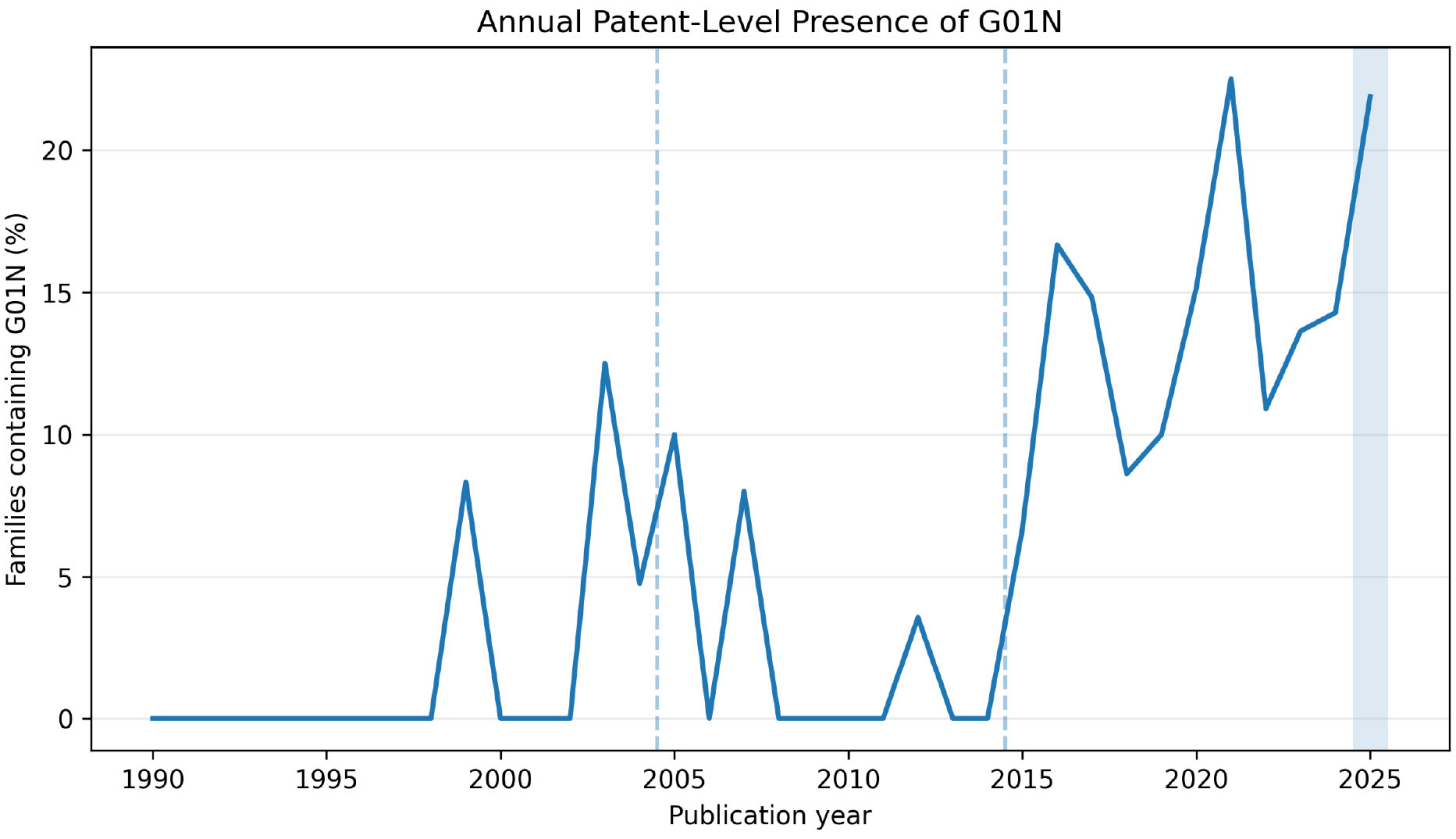
Annual patent-level presence of G01N.

A61K accounted for 15.9% of CPC tokens in 1990–2004, 14.7% in 2005–2014, and 8.9% in 2015–2025. The decline in relative share does not imply disappearance; rather, the technological portfolio broadened faster than medicinal-preparation classifications. By contrast, G01N's token share rose from 1.8% and 1.1% in the first two periods to 7.2% in the recent period. Patent-level G01N presence moved from 3.3% to 2.2% and then rose to 14.0% (Figure 4; Table 2). The recent-period level is approximately 4.2 times the first-period share and 6.3 times the second-period share.

**Table 2:** Domain share dynamics.

Period	A61K token share	G01N token share	G01N family presence
1990–2004	0.159	0.018	3.3%
2005–2014	0.147	0.011	2.2%
2015–2025	0.089	0.072	14.0%

These results establish analytical intensification, but they do not support the stronger claim that analytical chemistry is the single organizing backbone of the entire system. Its role is important and increasingly visible, yet it coexists with persistent processing nodes and other specialized connectors.

### 4.4 Bridging centrality under standardized edge weights

In the full-period Salton-weighted network, C11B (0.208) and A23L (0.164) occupy the two strongest bridging positions, followed by C05F (0.124), B02C (0.103), and A23D (0.091) (Table 3). G01N records a Salton betweenness of 0.078 and ranks ninth in the full-period network. Its Jaccard betweenness is higher (0.160), indicating that its bridging importance becomes more visible when shared-neighbor structure is emphasized.

**Table 3:** Leading CPC classes by Salton betweenness (full period).

CPC	Domain	Family frequency	Salton betweenness	Jaccard betweenness
C11B	Food processing and edible oils	192	0.208	0.106
A23L	Food processing and edible oils	151	0.164	0.172
C05F	Other technologies	15	0.124	0.161
B02C	Other technologies	14	0.103	0.113
A23D	Food processing and edible oils	143	0.091	0.104
C07H	Other technologies	3	0.089	0.120
Y02A	Sustainability and circularity	34	0.088	0.129
A61P	Pharmaceutical and nutraceutical	56	0.079	0.099
G01N	Analytical and validation	74	0.078	0.160
C05B	Other technologies	1	0.078	0.000

The temporal results are more revealing. G01N has no betweenness in the first period, remains modest in the second, and rises sharply in 2015–2025. In the recent network it ranks second under unweighted betweenness (0.143) and fifth under both Salton (0.143) and Jaccard (0.122) specifications. It is therefore a robust recent-period connector, but not the uniquely dominant bridge. Agreement among rankings is substantial: in the full corpus, Spearman correlations are 0.800 between unweighted and Salton betweenness, 0.916 between unweighted and Jaccard, and 0.837 between Salton and Jaccard.

### 4.5 Inside the analytical domain: G01N main groups

The 2015–2024 truncated window contains 58 G01N families among 433 patent families. Chromatographic separation (G01N 30/00) occurs in 25 G01N families (43.1%), and optical/spectrophotometric methods (G01N 21/00) occur in 22 (37.9%). Chemical or biological property determination (G01N 33/00) appears in 11 families (19.0%), electrochemical methods in four (6.9%), and magnetic-resonance methods in two (3.4%) (Table 4). The prevalence of chromatography and optical analysis supports the interpretation that the recent rise of G01N includes compositional and authentication-oriented technologies, although the large “other G01N” category cautions against equating the entire subclass with metabolomics or high-resolution instrumentation.

**Table 4:** Composition of G01N patent families at main-group level (2015–2024).

Main group	Analytical function	Families	Share of G01N families (%)	Share of all families (%)
G01N 33/00	Determination of chemical or biological properties of materials	11	19.0	2.5
G01N 30/00	Chromatographic separation techniques	25	43.1	5.8
G01N 21/00	Optical and spectrophotometric methods	22	37.9	5.1
G01N 24/00	Nuclear magnetic resonance and related methods	2	3.4	0.5
G01N 27/00	Electrochemical and other electrical methods	4	6.9	0.9
Other G01N groups	Remaining analytical and testing methods	33	56.9	7.6

Categories are non-mutually exclusive because a patent family may contain more than one G01N main group.

### 4.6 Visual evolution of the CPC network

**Figure 5:**
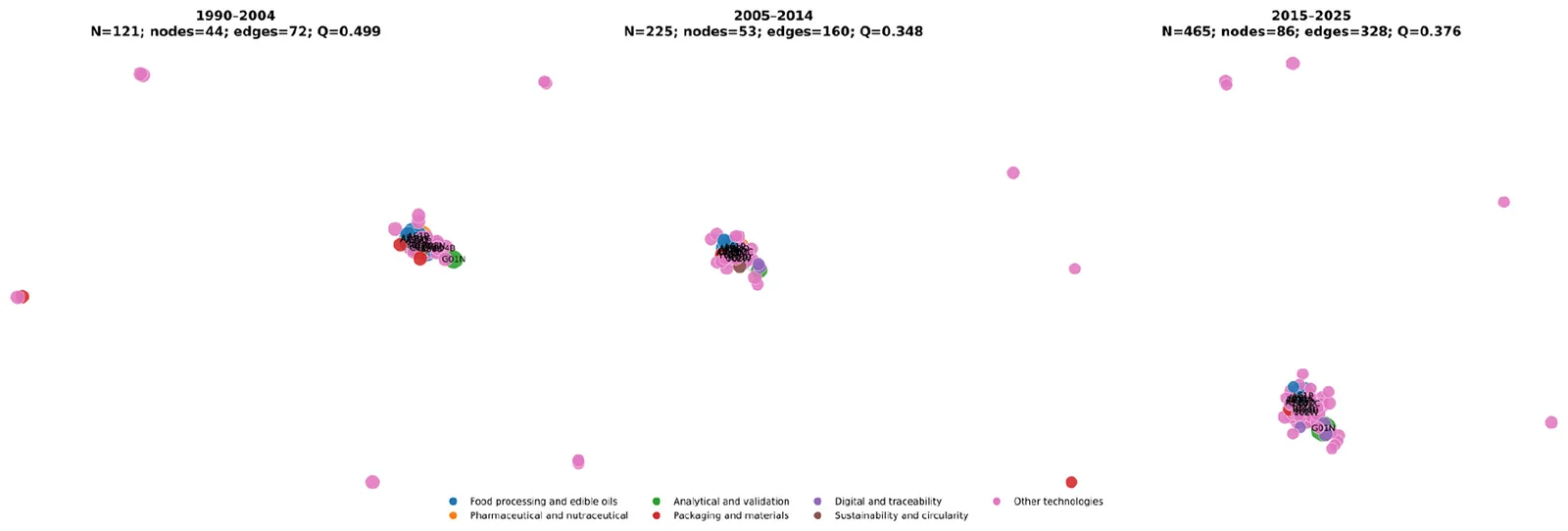
CPC co-classification networks across the three analytical periods. Node size reflects patent-family frequency; edges represent CPC co-occurrence. The maps are intended for structural comparison and do not imply literal movement of patent families between periods.

Figure 5 places the three period-specific networks side by side using a common visual logic. The first period is relatively small and fragmented. The second period shows a denser consolidated core, consistent with the decline in modularity and the rise in density. The recent period is substantially larger and contains more differentiated peripheral regions while preserving a connected core. G01N becomes visibly more prominent in the recent network, but the visual evidence also confirms the continuing structural importance of processing-related subclasses such as C11B, A23L, and A23D.

### 4.7 Robustness to normalization and the 2025 endpoint

The weighting robustness tests produce the same directional modularity pattern under raw, Salton/cosine, and Jaccard specifications: a decline from the first to the second period followed by a modest increase in the third. The centrality rankings are not identical, which is substantively informative, but their correlations are high and G01N remains a top-five recent-period bridge under both normalized measures.

Excluding 2025 reduces the recent-period sample from 465 to 433 families, nodes from 86 to 83, and edges from 328 to 316. Density changes from 0.0897 to 0.0929. Raw modularity changes from 0.3762 to 0.3808; Salton modularity from 0.5944 to 0.6068; and Jaccard modularity from 0.6762 to 0.6767. These differences are small, confirming that the interpretation of analytical diversification and selective re-specialization does not depend on the incomplete final year.

## 5 Discussion

### 5.1 Theoretical implications

The revised evidence changes the theoretical interpretation in an important way. VOO innovation clearly diversified, but the modularity trajectory does not describe a linear shift from processing to specialization and then reintegration. Instead, the earliest network is the most modular, the middle period is the most consolidated, and the recent period shows a modest return to modular differentiation within a much larger system. This pattern is consistent with selective re-specialization: new domains become more distinguishable without severing the connective processing core.

This result refines sectoral innovation-system theory by showing that technological expansion can alternate between consolidation and differentiation. Network growth alone cannot establish structural integration, and declining density in the most recent period should not be read as weakening innovation. Rather, it reflects the quadratic expansion of possible ties combined with uneven recombination across newly entering technological domains.

The analytical domain provides a second contribution. G01N's rapid recent growth and top-five normalized bridging position show that measurement, authentication, and compositional analysis increasingly connect otherwise distinct inventive activities. At the same time, the persistence of C11B and A23L as stronger full-period bridges cautions against a platform interpretation centered on a single analytical node. The more defensible conceptualization is a hybrid architecture in which processing remains foundational while analytical capabilities become increasingly important complements and connectors.

The main-group decomposition also narrows the chemical interpretation. Chromatographic and optical methods dominate the specified G01N categories, whereas NMR-related classifications remain uncommon. The evidence therefore supports analytical intensification broadly, especially separation and optical measurement, but does not justify treating high-resolution metabolomics as representative of the whole analytical patent domain.

### 5.2 Practical and policy implications

For firms, the results indicate that process competence remains strategically central, but it is no longer sufficient. Competitive R&D portfolios increasingly need chromatographic, spectrophotometric, quality-assurance, and authenticity capabilities. Because G01N is a significant recent bridge rather than the sole system backbone, firms should combine analytical investment with processing, formulation, sustainability, and packaging competencies.

For policymakers and standard-setting bodies, the results support investment in measurement infrastructure, validated methods, inter-laboratory comparability, and transparent compositional standards. Such infrastructure can lower coordination costs across processing, health-related formulation, and sustainability applications. Policy should nevertheless avoid assuming that regulation has already produced a fully integrated platform; the recent rise in modularity suggests that specialized technological communities remain important.

### 5.3 Limitations and future research

The study analyses formal patent classifications rather than market adoption, production performance, or scientific publications. CPC assignment depends on examiner practice, and four-character subclasses may combine heterogeneous technologies. The family corpus is also sensitive to Lens indexing and export date. The final analytical sample comprises 811 CPC-usable simple families from 1,993 exported records; future replication should archive the exact query, export date, and raw file alongside the code.

The temporal windows are analytically useful but not causal breakpoints. Alternative windows or rolling-network analysis could reveal whether the observed decline and subsequent rise in modularity occurred gradually or around specific regulatory and technological events. Future work should also disaggregate A61K and the residual G01N category, examine applicant and jurisdiction networks, and test whether the same consolidation–re-specialization pattern appears in other traditional agri-food sectors.

## 6 Conclusion

Using 811 simple patent families, this study documents substantial expansion in the technological architecture of virgin olive oil innovation between 1990 and 2025. The network grew from 44 nodes and 72 edges to 86 nodes and 328 edges, while analytical technologies became markedly more prevalent in the recent period.

The revised evidence does not support the stronger claim of a three-stage specialization–reintegration cycle or the claim that G01N is the highest-betweenness node across the full system. Instead, modularity indicates consolidation from the first to the second period followed by selective re-specialization in the third. G01N emerges as an important recent-period connector—second under unweighted and fifth under Salton and Jaccard betweenness—but processing-related classes remain structurally central.

The main theoretical implication is therefore one of hybrid evolution. VOO innovation has broadened beyond processing toward analytical, pharmaceutical, sustainability, and related domains, yet this diversification has not dissolved the processing core into a single integrated platform. Analytical capability is increasingly consequential, especially through chromatography and optical methods, but it functions as one component of a multi-centered innovation architecture. This more qualified interpretation is both more consistent with the standardized network evidence and more useful for R&D and policy decisions.

## Data Availability

The raw data supporting the conclusions of this article will be made available by the authors, without undue reservation. The cleaned family-level dataset and the analysis scripts are retained by the corresponding author and will be provided on reasonable request.
