# Supplementary material for: From processing-centered innovation to analytical diversification: network evolution in virgin olive oil patents
Source: Front Res Metr Anal. 2026 Aug 31;11:1873036. doi: 10.3389/frma.2026.1873036 (PMC13572592; doi:10.3389/frma.2026.1873036)
Supplement: Supplementary file 1 [file Supplementary_file_1.docx]

**Figure S1. Salton-Normalised CPC Network, 2015–2024.**
